# Association Between Periodontal Health Status and COVID-19 Severity: A Cross-Sectional Study

**DOI:** 10.3390/medicina62050858

**Published:** 2026-04-30

**Authors:** Mehmet Gümüş Kanmaz, Burcu Kanmaz, Pınar Ayvat, Timo Sorsa, Pınar Meriç Kantar, Nurcan Buduneli

**Affiliations:** 1 Department of Periodontology, Faculty of Dentistry, Izmir Tinaztepe University, 35390 Izmir, Turkey; 2 Department of Periodontology, Faculty of Dentistry, Dokuz Eylul University, 35340 Izmir, Turkey; 3 Department of Anesthesiology and Reanimation, Faculty of Medicine, Izmir Democracy University, 35290 Izmir, Turkey; 4 Department of Oral and Maxillofacial Diseases, Helsinki University Hospital, Helsinki University, 00014 Helsinki, Finland; 5 Department of Oral Diseases, Karolinska Institutet, Huddinge, 171 77 Stockholm, Sweden; 6 Department of Periodontology, Faculty of Dentistry, Ege University, 35040 Izmir, Turkey

**Keywords:** periodontitis, dental plaque, COVID-19, SARS-CoV-2, apache

## Abstract

*Background and Objectives*: The objective of this study was to investigate the relationship between clinical periodontal status and COVID-19 severity, including intensive care unit (ICU) admission and in-hospital mortality, in a cohort of hospitalized patients. *Materials and Methods*: This single-center, cross-sectional study included 44 patients with polymerase chain reaction-confirmed COVID-19 hospitalized at Buca Seyfi Demirsoy Training and Research Hospital, Izmir, Turkey, between August and December 2021. Of these, 32 (72.7%) were admitted to the ICU and 12 (27.3%) to the inpatient service. All participants underwent a full-mouth clinical periodontal examination to assess probing pocket depth (PPD), clinical attachment level (CAL), bleeding on probing (BoP), and plaque index (PI). Clinical data, demographics, comorbidities, and validated disease severity scores (GCS, APACHE II and SOFA) were extracted from electronic medical records, and a univariate logistic regression analysis was performed to identify factors associated with in-hospital mortality. *Results*: Patients admitted to the ICU (*n* = 32) were significantly older, had a higher prevalence of comorbidities, and showed higher CAL (*p* = 0.049) and PI (*p* < 0.001) values than those treated in the inpatient service. Deceased patients (*n* = 15) had a significantly higher PI than survivors (*p* = 0.024). In the univariate logistic regression analysis, APACHE II was the only variable significantly associated with in-hospital mortality (OR = 0.867, *p* = 0.003), however none of the periodontal parameters, including CAL and PI, showed a statistically significant association with mortality. *Conclusions*: Poorer periodontal findings, particularly higher CAL and PI values, were more frequently observed in patients requiring ICU care. However, periodontal parameters were not significantly associated with in-hospital mortality in univariate analysis. Given the cross-sectional design, small sample size, and lack of multivariable adjustment, these findings should be interpreted as unadjusted associations rather than evidence of an independent or causal relationship.

## 1. Introduction

Coronavirus disease (COVID-19), a respiratory illness caused by SARS-CoV-2, emerged in late 2019 and quickly became a global pandemic, causing over 778 million cases and 7.1 million deaths by August 2025 [1,2]. The disease manifests on a spectrum from mild symptoms, such as fever and fatigue, to critical conditions, such as acute respiratory distress and multi-organ failure [3,4]. This progression to severity is often triggered by a cytokine storm, an exaggerated host immune response that leads to hyperinflammation [5]. This state is marked by high levels of biomarkers, like interleukin-6 (IL-6), C-reactive protein (CRP), D-dimer, and ferritin, which are predictive of poor clinical outcomes and increased mortality rates [6,7].

The clinical course of COVID-19 is known to be significantly worsened by pre-existing systemic health conditions. Comorbidities such as hypertension, diabetes mellitus, cardiovascular disease, chronic respiratory disorders, and obesity, as well as immunosuppressive states from treatments like radiotherapy and chemotherapy, are strongly linked to a poorer prognosis and an increased risk of mortality [8,9,10]. However, beyond these established risk factors, there is a growing interest in the role of chronic inflammatory conditions, such as periodontitis, as potential modifiers of the clinical course of COVID-19 infection.

Periodontitis is a highly prevalent chronic inflammatory disease [11] that affects approximately 50% of adults worldwide with mild to moderate forms and 10% with severe manifestations [12,13]. It is characterized by the progressive destruction of the tooth-supporting apparatus, driven by dysbiotic subgingival biofilms and exacerbated by host immune responses [14,15]. Chronic systemic inflammation associated with periodontitis is reflected by elevated levels of cytokines, such as tumor necrosis factor-alpha (TNF-α), IL-1β, IL-6, and IL-10 [16,17], acute-phase reactants, such as CRP and ferritin [18], as well as active-matrix metalloproteinase (aMMP)-8 [19]. These inflammatory mediators significantly overlap with the pathophysiology of severe COVID-19, suggesting a potential relationship between the two diseases.

In light of these shared inflammatory pathways, this study aimed to investigate the associations between clinical periodontal parameters and COVID-19 severity, specifically focusing on intensive care unit (ICU) admission, in-hospital mortality, and disease severity scores, including the Glasgow Coma Scale (GCS), APACHE II, and SOFA, in a cohort of hospitalized patients.

## 2. Materials and Methods

This single-center, cross-sectional study was performed at Izmir Democracy University, Faculty of Medicine, following approval from the Ethics Committee (Approval No: 2021/08-05). The study was conducted in accordance with the Declaration of Helsinki and the STROBE guidelines.

### 2.1. Participants

Patients were recruited using consecutive sampling, enrolling all eligible individuals hospitalized at Buca Seyfi DEMIRSOY Training and Research Hospital from August 2021 to December 2021 who fulfilled the inclusion criteria. Patients aged ≥ 18 years with a reverse-transcription PCR-confirmed diagnosis of COVID-19, who presented with clinical symptoms and required hospitalization either in the dedicated COVID-19 inpatient service or the ICU, and who had at least one natural tooth were included in the study. The exclusion criteria comprised unconfirmed infection, complete edentulism, age < 18 years, inability or refusal to provide informed consent, and critical medical instability that precluded a safe full-mouth periodontal examination. Consent was obtained from the legal guardians or authorized representatives of the unconscious patients. Due to strict hospital infection control protocols and pandemic-related access restrictions, consecutive sampling was operationally limited, precluding continuous screening.

### 2.2. Clinical Records

Patient data were extracted from the electronic medical records. Baseline information, including demographics, vaccination status, and key comorbidities known to influence COVID-19 outcomes were recorded. Additionally, results for a panel of relevant blood parameters were collected to evaluate the inflammatory status and prognosis.

Furthermore, the clinical severity of each patient upon admission was documented. This involved recording the level of care, either COVID-19 inpatient service or ICU, and classifying the required respiratory support into one of three categories: no oxygen therapy, non-invasive oxygen delivery, or invasive mechanical ventilation.

In addition, for patients treated in the ICU, disease severity was quantified using the following indices extracted from the electronic medical records at the time of periodontal examination:

• Glasgow Coma Scale (GCS): Neurological responsiveness was quantified using the GCS, which assigns separate sub-scores for eye opening (E, 1–4), verbal response (V, 1–5), and motor response (M, 1–6). The three components were recorded contemporaneously and summed to yield a total GCS ranging from 3 (no eye, verbal, or motor response) to 15 (normal consciousness), with higher values indicating better neurological function [20].
• Acute Physiology and Chronic Health Evaluation II (APACHE II): To measure illness severity, the APACHE II score was calculated within 24 h of ICU admission. Based on the original algorithm by Knaus et al. [21], this score combines 12 physiological variables with the patient’s age and chronic health status. The final score (0–71) corresponds to the acuity of the illness, with higher values predicting an increased risk of in-hospital mortality.
• Sequential Organ Failure Assessment (SOFA): The SOFA score was calculated on the day of ICU admission to evaluate multi-organ dysfunction. This tool assesses six organ systems (respiration, coagulation, liver, cardiovascular, central nervous system, and renal), grading the dysfunction of each system on a scale from 0 to 4 [22].

### 2.3. Clinical Periodontal Examination

Prior to recruitment, two investigators (BK and MGK) were calibrated on ten COVID-19–negative volunteers, yielding high inter-examiner agreement (κ = 0.91 for categorical variables; ICC = 0.93 for continuous variables).

To ensure safety in the infectious disease setting, infection control was managed using appropriate personal protective equipment (PPE), including N95 respirators, full-body gowns, gloves, face shields, and shoe covers, in accordance with hospital protocols for COVID-19 patient interactions. Examinations were timed to avoid interference with medical care, with assessments scheduled in coordination with the healthcare team during periods of patient stability and minimal clinical interventions, thereby minimizing the risks to both patients and examiners.

The two calibrated examiners (BK, MGK) performed full-mouth clinical periodontal charting at six sites per tooth with a manual UNC-15 probe (Hu-Friedy, Chicago, IL, USA), documenting visible plaque (Silness–Löe Plaque Index), bleeding on probing (BoP) (Ainamo & Bay), probing pocket depth (PPD), clinical attachment level (CAL), and the number of missing teeth.

Patients were classified as healthy, gingivitis [23] or periodontitis [24], based on the current classification of periodontal diseases and conditions [25]. Gingival health was defined as <10% bleeding sites with PPD ≤ 3 mm. Gingivitis was defined as ≥10% bleeding sites with PPD ≤ 3 mm, and periodontitis was defined as a detectable interdental CAL at ≥2 nonadjacent teeth, or buccal or oral CAL ≥ 3 mm with pocketing ≥ 3 mm at ≥2 teeth when not ascribed to non-periodontitis-related causes.

### 2.4. aMMP-8 Point-of-Care Testing

Detection of aMMP-8 was carried out with lateral-flow point-of-care immunoassay (PerioSafe, Dentognostics GmbH, Jena, Germany) according to the manufacturer’s instructions [26,27]. This procedural step was limited to conscious patients, as the sample collection protocol required the patient to actively rinse his/her mouth with a solution and then expectorate the entire rinse into a sterile collection cup, actions that are not feasible for unconscious or intubated patients.

### 2.5. Sample Size Determination and Statistical Analysis

Using G*Power 3.1 (effect size 0.50, α = 0.05, power = 95%), the minimum required sample was 34 participants; to allow for attrition, the target enrolment was ≥40. Statistical analyses were conducted using IBM SPSS Statistics version 25 (IBM Corp., Armonk, NY, USA). The Shapiro–Wilk test was used to assess the normality of the data distribution. For normally distributed continuous data, an independent-samples t-test was employed for comparisons, while the Mann–Whitney U test was used for non-normally distributed data. Categorical variables were analyzed using the Chi-square Test, and Spearman’s correlation coefficient was used to explore associations between continuous variables. To control for Type I errors across multiple comparisons, a Bonferroni correction was applied. Given the limited sample size and the small number of mortality events, multivariable logistic regression was considered statistically unreliable and at high risk of overfitting. Therefore, univariate binary logistic regression analyses were used to identify factors associated with in-hospital mortality, and the findings were interpreted as exploratory and unadjusted associations. Statistical significance was set at *p* < 0.05.

## 3. Results

During the 5-month recruitment period, strict hospital infection control protocols and pandemic-related access restrictions severely limited the pool of patients available for initial eligibility screening to 93 individuals. From this accessible cohort, substantial exclusions were necessary based on our established criteria and safety protocols. Specifically, 34 patients (or their authorized proxies) declined participation, and 3 patients were excluded due to complete edentulism. Additionally, 12 severe cases in the ICU were excluded because critical medical instability prevented a safe full-mouth clinical periodontal examination without disrupting life-saving emergency medical care. Consequently, the final analyzed cohort consisted of 44 patients. The primary outcome was the association between clinical periodontal status and COVID-19 severity, defined mainly by hospitalization type (ICU vs. inpatient service). Secondary outcomes included the association of periodontal parameters with in-hospital mortality, their correlations with disease severity scores (GCS, APACHE II, and SOFA) and laboratory parameters, and the exploratory evaluation of aMMP-8 test positivity.

The mean age of the cohort was 65.79 ± 15.04 years, with a predominance of male participants (72.7%). Most patients (72.7%) were admitted to the ICU, whereas 27.3% received treatment in the inpatient service. Regarding respiratory support, 29.5% were intubated, 47.7% received oxygen via face mask, and 22.7% did not require supplemental oxygen. Comorbidities were present in 63.6% of the cohort, most commonly hypertension (40.9%) and diabetes mellitus (20.5%). Vaccination status varied: 36.4% were unknown, 18.2% were unvaccinated, and the remainder received two or more doses of CoronaVac (Sinovac Life Sciences Co., Ltd., Beijing, China) and/or BNT162b2 (Comirnaty^®^, Pfizer–BioNTech, Mainz, Germany and New York, USA). The overall mortality rate was 34.1% (*n* = 15).

The mean number of missing teeth was 15.05 ± 9.65. The mean PPD and CAL were 2.79 ± 0.64 mm and 3.80 ± 1.05 mm, respectively. BoP occurred in 36.11% ± 37.32 of the sites. The mean PI was 86.55 ± 26.57, indicating extensive dental plaque accumulation. Notably, several ICU patients exhibited maximum PI scores (100%), suggesting plaque presence on all evaluated tooth surfaces.

aMMP-8 testing was performed only in a small subset of conscious and cooperative patients who were able to complete the mouth-rinse procedure. Within this subgroup, all tested ICU patients were positive for aMMP-8, compared with 66.7% of tested inpatient-service patients; however, this difference was not statistically significant (*p* = 0.576). These findings should be interpreted cautiously and should not be considered representative of the entire ICU cohort.

In the ICU subgroup (*n* = 32), the mean GCS score was 10.03 ± 5.20, the APACHE II score was 18.22 ± 11.40, and the SOFA score was 7.38 ± 4.72. The mortality rate in the ICU group was 46.9%. Patients in the ICU were significantly older than those in the inpatient service (median 67.00 vs. 55.00 years; *p* = 0.011). Respiratory support requirements differed markedly (*p* < 0.001): all intubated patients were in the ICU, whereas 83.3% of inpatient service patients required no oxygen therapy. ICU patients had a significantly higher prevalence of comorbidities (87.5% vs. 0%, *p* < 0.001) and mortality rate (46.9% vs. 0%, *p* = 0.003). Vaccination status also differed (*p* = 0.003), with a greater proportion of inpatient service patients being unvaccinated (50% vs. 6.3%). Periodontal analysis revealed significantly higher CAL (median 3.00 mm vs. 2.73 mm; *p* = 0.049) and PI (median 100% vs. 60%; *p* < 0.001) in ICU patients, while other parameters, PPD, missing teeth, BoP, and aMMP-8 test, showed no significant difference (Table 1).

Analysis within the ICU subgroup revealed no statistically significant differences in periodontal parameters between survivors (*n* = 17) and non-survivors (*n* = 15). Specifically, CAL, PPD, PI, and the number of missing teeth were comparable between the two groups (*p* > 0.05). BoP tended to be higher among non-survivors than survivors (median 60.00 vs. 0.00), although this difference did not reach statistical significance (*p* = 0.069).

When comparing deceased patients (*n* = 15) to survivors (*n* = 29), no significant differences were observed regarding baseline age (*p* = 0.425) or sex (*p* = 0.621). Mortality was strongly associated with the need for invasive respiratory support, as 80.0% of the deceased patients were intubated compared to only 3.4% of the survivors (*p* < 0.001). While comorbidity prevalence (*p* = 0.185) and vaccination status (*p* = 0.140) did not differ significantly between the groups, periodontal evaluation revealed that the PI was significantly elevated among deceased patients (median 100.00 [IQR: 100.00–100.00] vs. 100.00 [IQR: 65.25–100.00]; *p* = 0.024). Conversely, all other assessed periodontal measures, including PPD (*p* = 0.766), CAL (*p* = 0.435), missing teeth (*p* = 0.833), BoP (*p* = 0.164), and aMMP-8 test results (*p* = 0.733), showed no statistically significant differences between survivors and non-survivors (Table 2).

Based on periodontal diagnosis, the entire cohort consisted of patients with either gingivitis (*n* = 32) or periodontitis (*n* = 12); no participants were classified as periodontally healthy. Comparison of patients with gingivitis versus periodontitis, no statistically significant differences were found between the two groups regarding age, hospitalization type, oxygen requirement, comorbidities, or survival outcomes. However, the periodontitis group showed significantly greater PPD (*p* < 0.001), CAL (*p* < 0.001), and BoP (*p* = 0.014), confirming the diagnostic differentiation (Table 3).

Univariate logistic regression identified the APACHE II score as the only variable significantly associated with in-hospital survival (*p* = 0.003). A higher APACHE II score was associated with lower odds of survival (OR = 0.867). No other tested variables, CAL (*p* = 0.488), PI (*p* = 0.116), age (*p* = 0.415) or the presence of comorbidities (*p* = 0.114), reached statistical significance in the model. (Table 4).

Spearman’s correlation revealed that age was positively correlated with the number of missing teeth (*r* = 0.537, *p* < 0.001). The PI moderately correlated with age (*r* = 0.358, *p* = 0.022) and number of missing teeth showed a positive correlation with procalcitonin levels (*r* = 0.321, *p* = 0.036). SOFA score was negatively correlated with PPD (*r* = –0.356, *p* = 0.045). After Bonferroni correction, only the age–missing teeth correlation remained significant (Table 5).

## 4. Discussion

The present study, involving 44 hospitalized patients with COVID-19, showed that patients in the ICU group had significantly higher CAL and PI values than those treated in the inpatient service. The ICU group was also older and had a higher prevalence of comorbidities. Although PI was significantly higher among deceased patients than among survivors, no other clinical periodontal parameters differed significantly according to survival status. However, given the cross-sectional design and the use of univariate logistic regression, these findings should be interpreted as unadjusted associations rather than evidence of independent or causal relationships. In the univariate logistic regression analysis, APACHE II was the only variable significantly associated with in-hospital mortality, whereas periodontal parameters, including CAL and PI, were not significantly associated with mortality. In the correlation analysis, after Bonferroni correction, only the positive association between age and the number of missing teeth remained statistically significant.

The finding that patients in the ICU group had higher CAL and PI values than those treated in the inpatient service is directionally consistent with the case-control study by Marouf et al. [28], who reported an adjusted association between radiographically defined periodontitis and adverse COVID-19 outcomes, including ICU admission. However, the two studies are not directly comparable. Marouf et al. evaluated periodontitis retrospectively from radiographic bone loss and applied multivariable adjustment, whereas the present study was based on direct full-mouth clinical periodontal examination and the ICU-group comparison was based on unadjusted between-group analyses. Moreover, the ICU group in the present cohort was older and had a substantially higher prevalence of comorbidities, which may have influenced the observed periodontal differences.

Comparison with Macherla et al. [29] also supports a cautious interpretation. In that study, periodontitis was associated with greater COVID-19 severity, whereas plaque and calculus scores did not differ significantly between severity groups and the mortality-related findings were limited. In the present cohort, PI was higher in the ICU group and among deceased patients; however, our internal comparison between gingivitis and periodontitis groups did not show significant differences in ICU admission or survival, and periodontal parameters were not significantly associated with mortality in the univariate logistic regression analysis. Costa et al. [30], by contrast, reported that periodontitis remained associated with ICU admission, critical symptoms, and risk of death after adjustment for age and comorbidities in a larger hospitalized cohort. Taken together, these studies suggest that poorer periodontal findings may be more frequently observed in patients with more severe COVID-19-related clinical status, but the strength and independence of any association with mortality remain uncertain and appear to be influenced by study design, sample size, timing of periodontal assessment, and the degree of statistical adjustment.

The findings of Anand et al. [31] are relevant to the broader relationship between oral health and COVID-19, although their study addressed COVID-19 positivity rather than disease severity or in-hospital mortality among hospitalized patients. Anand et al. [31] reported that higher plaque scores, gingivitis, greater CAL, and severe periodontitis were more frequent in COVID-19-positive patients than in COVID-19-negative controls. Their logistic regression analysis revealed that individuals with a mean plaque score ≥1 had over seven times the odds of being in the COVID-19 positive group. This aligns with our observation that the PI was significantly higher in deceased patients, however this should be interpreted with caution. Given the cross-sectional design, the older and more comorbid ICU population, plaque accumulation may have reflected dependency, reduced self-care capacity, or hospitalization-related limitations in oral hygiene rather than a pre-existing prognostic factor. However, another mechanism underlying this association may be explained by increased dental plaque levels that create an environment for the oral carriage of respiratory pathogens, which in turn could cause COVID-19-related complications. This connection has been observed in other respiratory conditions; periodontitis has been linked to both chronic obstructive pulmonary disease and pneumonia through the direct aspiration of oral pathogens into the lungs or by the alteration of mucous surfaces in the respiratory tract, which promotes the adhesion and invasion of pathogens [32,33,34,35].

From a pathophysiological perspective, Moradi Haghgoo et al. [36] reported that severe periodontitis was associated with more severe COVID-19 and with higher salivary and serum IL-6 levels, while a separate related analysis by the same group [37] found associations between the interaction of periodontitis and COVID-19 severity and selected hematologic parameters, particularly white blood cell and platelet counts. These findings support the biological plausibility of a link between periodontal inflammation and adverse COVID-19 outcomes. However, the present study did not assess IL-6 or those specific hematologic indices. Moreover, although some correlations were observed between periodontal parameters and systemic markers, none remained statistically significant after Bonferroni correction. Therefore, while the findings of Moradi Haghgoo et al. [36] provide supportive pathophysiological context, our data do not support a mechanistic interpretation regarding the contribution of periodontal findings to systemic inflammatory burden in this cohort. Similarly, no significant associations were found between aMMP-8 results and clinical outcomes, survival status, or other periodontal variables, further supporting a cautious interpretation of this exploratory analysis.

A systematic review and meta-analysis by Al-Maweri et al. [38] also supports a possible association between periodontal disease and adverse COVID-19 outcomes. In their pooled analysis, periodontal disease was associated with higher odds of severe symptoms and ICU admission, whereas the association with mortality was not statistically significant when patients with periodontal disease were compared with those with healthy periodontium. They also reported that severe periodontal disease, compared with mild periodontal disease, was associated with worse COVID-19 outcomes, including ICU admission and mortality. In the present study, ICU patients likewise showed higher CAL and PI values than those treated in the inpatient service, which is broadly consistent with the severity-related findings of that review. However, periodontal parameters were not significantly associated with mortality in our univariate regression analysis. Therefore, while the findings of Al-Maweri et al. [38] provide supportive context for an association between poorer periodontal status and more severe COVID-19-related clinical presentation, our results do not support an independent interpretation regarding mortality. This cautious interpretation is further justified by the substantial methodological heterogeneity highlighted in their review, including variability in periodontal assessment methods, with studies relying on clinical examination, self-report, or radiographs. Although the present study used direct full-mouth clinical periodontal examination, its cross-sectional design and limited sample size still warrant cautious interpretation.

The present study has several key strengths that enhanced the validity of its findings. The primary strength is the use of a direct, full-mouth clinical periodontal examination performed by two calibrated examiners on all participants. This rigorous, standardized approach to data collection avoids recall bias and inaccuracies associated with self-reported data or the limitations of retrospective radiographic analysis, providing a more reliable assessment of the patients’ current periodontal and oral hygiene status. Furthermore, the comprehensive collection of clinical data, including validated severity scores like APACHE II and SOFA, allowed for a robust analysis that could differentiate the influence of acute systemic illness from chronic periodontal conditions.

Despite these strengths, several limitations should be acknowledged. First, this was a single-center cross-sectional study with a relatively small sample size (*n* = 44), which limits generalizability and increases the possibility of type II error, particularly for subgroup comparisons and mortality analyses. An important methodological distinction should also be emphasized regarding statistical power. The power reported in this study applies to the primary comparison between periodontal status and hospitalization type, not to the logistic regression analyses for mortality. Because the mortality analysis was based on a relatively small number of events, the ability to detect moderate associations, particularly for periodontal variables, was limited. Accordingly, the lack of statistically significant associations for CAL and PI should be interpreted cautiously, as these null findings may partly reflect limited statistical power rather than true absence of association. This issue may be particularly relevant for PI, which showed a marked ceiling effect in the present cohort, thereby reducing the variability available for regression modeling. Second, the mortality analysis was limited to univariate logistic regression. Although a multivariable logistic regression analysis was also performed, the model was statistically unstable and prone to overfitting because of the limited sample size and low event count. Therefore, the findings were interpreted primarily as exploratory and unadjusted associations. This is particularly relevant given the marked imbalance between the ICU and inpatient-service groups, as ICU patients were older, had substantially more comorbidities, and accounted for all deaths in the cohort. Third, the cross-sectional design precludes conclusions about temporality or causality. In particular, the higher plaque index observed in ICU patients and in deceased patients may reflect reverse causation, as critically ill, intubated, or sedated patients are often unable to maintain oral hygiene during hospitalization. Fourth, the exclusion of edentulous patients may have introduced selection bias, as these individuals may represent those with the poorest historical oral health. Fifth, vaccination status was unknown in a substantial proportion of participants, which further complicates interpretation because vaccination is an important modifier of COVID-19 severity. This missing information may have introduced residual confounding, as differences in vaccination coverage between groups could have influenced ICU admission, mortality, and disease severity independently of periodontal status. Finally, the aMMP-8 analysis was limited to a very small subset of conscious and cooperative patients and showed no significant associations with clinical outcomes.

## 5. Conclusions

In this cohort of hospitalized COVID-19 patients, poorer periodontal findings, particularly higher CAL and PI values, were more frequently observed in patients with more severe clinical presentation. However, given the cross-sectional design, the small sample size, and the lack of multivariable adjustment, these findings should be interpreted as unadjusted associations rather than evidence of an independent or causal relationship. In the present dataset, APACHE II was the only variable significantly associated with in-hospital mortality in univariate analysis. Overall, periodontal status may reflect underlying patient vulnerability, but larger prospective studies with adequate multivariable adjustment are needed to clarify its clinical significance in COVID-19 outcomes.

## Figures and Tables

**Table 1:** Comparison of demographic data and periodontal parameters between units.

	Intensive Care Unit (*n* = 32)	Inpatient Service (*n* = 12)	*p*-Value
Age (*n* = 44), median (Q1; Q3)	67.00 (58.00; 78.00)	55.00 (44.00; 60.00)	0.011 *
Gender (*n* = 44), *n* (%)			0.579
Female	9 (28.1%)	3 (25.0%)	
Male	23 (71.9%)	9 (75.0%)	
Oxygen requirement (*n* = 44), *n* (%)			0.000 †
Intubated	13 (40.6%)	0 (0.0%)	
Face mask	19 (59.4%)	2 (16.7%)	
Not required	0 (0.0%)	10 (83.3%)	
Vaccination status (*n* = 44), *n* (%)			0.003 †
Unknown	16 (50.0%)	0 (0.0%)	
Not vaccinated	2 (6.3%)	6 (50.0%)	
2 CoronaVac	5 (15.6%)	1 (8.3%)	
2 BNT162b2	2 (6.3%)	2 (16.7%)	
2 CoronaVac+ 1 BNT162b2	2 (6.3%)	0 (0.0%)	
3 CoronaVac	5 (15.6%)	3 (25.0%)	
Comorbidities (*n* = 44), *n* (%)			0.000 †
Absent	4 (12.5%)	12 (100.0%)	
Present	28 (87.5%)	0 (0.0%)	
Type of comorbidities (*n* = 44), *n* (%)			
Hypertension	18 (56.25%)	0 (0.0%)	
Diabetes mellitus	9 (28.13%)	0 (0.0%)	
Chronic obstructive pulmonary disease	6 (18.75%)	0 (0.0%)	
Coronary artery disease	4 (12.5%)	0 (0.0%)	
Chronic kidney failure	3 (9.38%)	0 (0.0%)	
Parkinson disease	1 (3.13%)	0 (0.0%)	
Cerebrovascular disease	2 (6.25%)	0 (0.0%)	
Asthma	2 (6.25%)	0 (0.0%)	
Alzheimer’s disease	2 (6.25%)	0 (0.0%)	
Rheumatoid arthritis	1 (3.13%)	0 (0.0%)	
Obesity	1 (3.13%)	0 (0.0%)	
Chronic bronchitis	1 (3.13%)	0 (0.0%)	
Down syndrome	1 (3.13%)	0 (0.0%)	
Heart failure	2 (6.25%)	0 (0.0%)	
Breast cancer	1 (3.13%)	0 (0.0%)	
Sleep apnea	1 (3.13%)	0 (0.0%)	
Survival (*n* = 44), *n* (%)			0.003 †
Deceased	15 (46.9%)	0 (0.0%)	
Survived	17 (53.1%)	12 (100.0%)	
Missing teeth (*n* = 44), median (Q1; Q3)	17.50 (5.25; 26.50)	7.50 (5.25; 19.50)	0.273
Probing depth (*n* = 44) (mm), median (Q1; Q3)	2.79 (2.30; 3.00)	2.73 (2.26; 3.25)	0.579
Clinical attachment level (*n* = 44) (mm), median (Q1; Q3)	3.00 (2.74; 4.67)	2.73 (2.26; 3.25)	0.049 ‡
Bleeding on probing (*n* = 44), median (Q1; Q3)	25.00 (0.00; 70.00)	20.00 (5.00; 90.00)	0.509
Plaque index (*n* = 44), median (Q1; Q3)	100.00 (100.00; 100.00)	60.00 (18.00; 95.00)	0.000 ‡
aMMP-8 test (+/−) (*n* = 15), *n* (%)	3 (100.0%)/0 (0.0%)	8 (66.7%)/4 (33.3%)	0.576

* *p* < 0.05 Independent Samples T-Test; † *p* < 0.05 Chi-Square Test; ‡ *p* < 0.05 Mann-Whitney U Test.

**Table 2:** Comparison of demographic data and periodontal status between survival status.

	Deceased (*n* = 15)	Survived (*n* = 29)	*p*-Value
Age (*n* = 44), mean ± SD	64.57 ± 15.79	60.00 ± 14.42	0.425
Gender (*n* = 44), *n* (%)			0.621
Female	4 (26.7%)	8 (27.6%)	
Male	11 (73.3%)	21 (72.4%)	
Oxygen requirement (*n* = 44), *n* (%)			0.000 *
Intubated	12 (80.0%)	1 (3.4%)	
Face mask	3 (20.0%)	18 (62.0%)	
Not required	0 (0.0%)	10 (34.4%)	
Vaccination status (*n* = 44), *n* (%)			0.140
Unknown	9 (60.0%)	7 (24.1%)	
Not vaccinated	1 (6.7%)	7 (24.1%)	
2 CoronaVac	2 (13.3%)	4 (13.8%)	
2 BNT162b2	0 (0.0%)	4 (13.8%)	
2 CoronaVac+ 1 BNT162b2	0 (0.0%)	2 (6.9%)	
3 CoronaVac	3 (20.0%)	5 (17.2%)	
Comorbidities (*n* = 44), *n* (%)			0.185
Absent	3 (20.0%)	13 (44.8%)	
Present	12 (80.0%)	16 (55.2%)	
Type of comorbidities (*n* = 44), *n* (%)			
Hypertension	6 (40.0%)	12 (41.4%)	
Diabetes mellitus	3 (20.0%)	6 (20.7%)	
Chronic obstructive pulmonary disease	4 (26.7%)	2 (6.9%)	
Coronary artery disease	3 (20.0%)	1 (3.4%)	
Chronic kidney failure	2 (13.3%)	1 (3.4%)	
Parkinson disease		1 (3.4%)	
Cerebrovascular disease		2 (6.9%)	
Asthma		2 (6.9%)	
Alzheimer’s disease	2 (13.3%)		
Rheumatoid arthritis	1 (6.7%)		
Obesity		1 (3.4%)	
Chronic bronchitis	1 (6.7%)		
Down syndrome	1 (6.7%)		
Heart failure	1 (6.7%)	1 (3.4%)	
Breast cancer		1 (3.4%)	
Sleep Apnea	1 (6.7%)		
Missing teeth (*n* = 44), median (Q1; Q3)	18.50 (4.75; 27.00)	9.50 (5.75; 18.50)	0.833
Probing pocket depth (*n* = 44) (mm), median (Q1; Q3)	2.72 (2.11; 3.09)	2.80 (2.64; 4.18)	0.766
Clinical attachment level (*n* = 44) (mm), median (Q1; Q3)	3.05 (2.64; 4.18)	2.95 (2.63; 4.27)	0.435
Bleeding on probing (*n* = 44), median (Q1; Q3)	62.50 (7.50; 81.00)	15.00 (0.00; 60.00)	0.164
Plaque index (*n* = 44), median (Q1; Q3)	100.00 (100.00; 100.00)	100.00 (65.25; 100.00)	0.024 †
aMMP-8 test (+/−) (*n* = 15), *n* (%)	1 (100%)/0 (0.0%)	10 (71.4%)/4 (28.6%)	0.733

* *p* < 0.05 Chi-Square Test; † *p* < 0.05 Mann-Whitney U Test.

**Table 3:** Comparison of demographic data between different periodontal status subgroups.

	Gingivitis (*n* = 32)	Periodontitis (*n* = 12)	*p*-Value
Age (*n* = 44), mean ± SD	62.43 ± 16.21	63.55 ± 12.80	0.919
Gender (*n* = 44), *n* (%)			0.579
Female	9 (28.1%)	3 (25.0%)	
Male	23 (71.9%)	9 (75.0%)	
Hospital admission (*n* = 44), *n* (%)			0.421
Intensive care unit	24 (75.0%)	8 (66.7%)	
Inpatient service	8 (25.0%)	4 (33.3%)	
Oxygen requirement (*n* = 44), *n* (%)			0.589
Intubated	10 (31.3%)	3 (25.0%)	
Face mask	16 (50.0%)	5 (41.7%)	
Not required	6 (18.8%)	4 (33.3%)	
Vaccination status (*n* = 44), *n* (%)			0.598
Unknown	12 (37.5%)	4 (33.3%)	
Not vaccinated	5 (15.6%)	3 (25.0%)	
2 CoronaVac	3 (9.4%)	3 (25.0%)	
2 BNT162b2	3 (9.4%)	1 (8.3%)	
2 CoronaVac+ 1 BNT162b2	2 (6.3%)	0 (0.0%)	
3 CoronaVac	7 (21.9%)	1 (8.3%)	
Survival (*n* = 44), *n* (%)			0.621
Deceased	11 (34.4%)	4 (33.3%)	
Survived	21 (65.6%)	8 (66.7%)	
Comorbidities (*n* = 44), *n* (%)			0.732
Absent	11 (34.4%)	5 (41.7%)	
Present	21 (65.6%)	7 (58.3%)	
Type of comorbidities (*n* = 44), *n* (%)			
Hypertension	15 (46.9%)	3 (25.0%)	
Diabetes mellitus	7 (21.9%)	2 (16.7%)	
Chronic obstructive pulmonary disease	5 (15.6%)	1 (8.3%)	
Coronary artery disease	4 (12.5%)		
Chronic kidney failure	2 (6.2%)	1 (8.3%)	
Parkinson disease	1 (3.1%)		
Cerebrovascular disease	2 (6.2%)		
Asthma	2 (6.2%)		
Alzheimer’s disease	2 (6.2%)		
Rheumatoid arthritis	1 (3.1%)		
Obesity	1 (3.1%)	1 (8.3%)	
Chronic bronchitis	1 (3.1%)		
Down syndrome		1 (8.3%)	
Heart failure	1 (3.1%)	1 (8.3%)	
Breast cancer		1 (8.3%)	
Sleep Apnea		1 (8.3%)	
Missing teeth (*n* = 44), median (Q1; Q3)	8.00 (5.00; 24.75)	18.00 (6.00; 20.00)	0.615
Probing pocket depth (*n* = 44) (mm), median (Q1; Q3)	2.67 (2.23; 2.82)	3.38 (2.75; 4.07)	0.000 *
Clinical attachment level (*n* = 44) (mm), median (Q1; Q3)	2.77 (2.33; 3.00)	4.67 (3.38; 5.17)	0.000 *
Bleeding on probing (*n* = 44), median (Q1; Q3)	12.50 (0.00; 50.00)	65.00 (60.00; 100.00)	0.014 *
Plaque index (*n* = 44), median (Q1; Q3)	100.00 (69.00; 100.00)	100.00 (85.00; 100.00)	0.650

* *p* < 0.05 Mann-Whitney U Test.

**Table 4:** Univariate Logistic Regression Analyses of Factors Associated with In-Hospital Mortality.

Independent Variable	B (Coefficient)	S.E.	Wald	*p*-Value	OR (Exp(B))	95% C.I. for OR
Periodontal parameters						
Clinical attachment level (CAL)	−0.209	0.301	0.481	0.488	0.812	0.450–1.463
Plaque index (PI)	−0.060	0.038	2.806	0.116	0.942	0.875–1.015
Demographic and clinical parameters						
Age	−0.018	0.022	0.664	0.415	0.982	0.940–1.026
Comorbidities	−1.179	0.746	2.498	0.114	0.308	0.071–1.327
APACHE II score	−0.143	0.048	8.736	**0.003**	0.867	0.788–0.953

S.E. = Standard Error; df = degrees of freedom; OR = Odds Ratio; C.I. = Confidence Interval. Dependent Variable: Survival Status (0 = Deceased, 1 = Survived). Statistically significant *p*-values (<0.05) are shown in bold.

**Table 5:** Correlation Between Periodontal, Clinical, and Laboratory Parameters.

		Probing Pocket Depth	Clinical Attachment Level	Bleeding on Probing	Plaque Index	Missing Teeth
Age	Correlation	−0.053	0.242	0.104	0.358	0.537
	*p* value	0.743	0.127	0.517	0.022	0.000 *
GCS	Correlation	0.087	−0.151	−0.380	−0.011	−0.079
	*p* value	0.637	0.409	0.032	0.951	0.669
APACHE II score	Correlation	−0.251	0.152	0.281	0.082	0.282
	*p* value	0.166	0.406	0.119	0.657	0.119
SOFA score	Correlation	−0.356	0.149	0.221	0.059	0.296
	*p* value	0.045	0.415	0.224	0.750	0.100
Albumin	Correlation	0.048	0.030	−0.200	−0.193	−0.635
	*p* value	0.889	0.931	0.555	0.570	0.036
LDH	Correlation	0.144	0.093	−0.029	0.514	−0.278
	*p* value	0.673	0.786	0.933	0.106	0.408
AST	Correlation	−0.658	−0.689	−0.001	−0.327	0.271
	*p* value	0.028	0.019	0.997	0.326	0.420
ALT	Correlation	−0.383	−0.365	−0.348	−0.397	−0.073
	*p* value	0.245	0.270	0.294	0.227	0.832
Glucose	Correlation	0.264	0.229	−0.128	0.478	−0.162
	*p* value	0.434	0.498	0.709	0.137	0.633
Troponin	Correlation	0.049	−0.065	−0.212	0.224	0.393
	*p* value	0.863	0.817	0.448	0.423	0.148
Ferritin	Correlation	−0.029	0.274	0.251	0.270	0.488
	*p* value	0.853	0.076	0.104	0.079	0.001
Creatinine	Correlation	−0.241	0.243	0.016	0.031	0.155
	*p* value	0.124	0.120	0.922	0.845	0.326
D-Dimer	Correlation	0.058	0.155	−0.010	0.196	0.164
	*p* value	0.713	0.320	0.950	0.209	0.293
Procalcitonin	Correlation	−0.178	0.341	0.076	0.127	0.321
	*p* value	0.252	0.025	0.628	0.418	0.036
CRP	Correlation	−0.356	0.137	0.053	−0.051	0.141
	*p* value	0.046	0.455	0.775	0.783	0.443

* Spearman Correlation is significant at 0.000 level (2-tailed) (Bonferroni corrected).

## Data Availability

Data are available upon reasonable request from the corresponding author.

## Abbreviations

The following abbreviations are used in this manuscript:

ALT	Alanine Aminotransferase
aMMP-8	Active Matrix Metalloproteinase-8
APACHE II	Acute Physiology and Chronic Health Evaluation II
AST	Aspartate Aminotransferase
BNT162b2	Pfizer–BioNTech COVID-19 vaccine
BoP	Bleeding on Probing
CAL	Clinical Attachment Level
COVID-19	Coronavirus Disease 2019
CRP	C-reactive Protein
GCS	Glasgow Coma Scale
ICC	Intraclass Correlation Coefficient
ICU	Intensive Care Unit
IL	Interleukin
LDH	Lactate Dehydrogenase
OR	Odds Ratio
PCR	Polymerase Chain Reaction
PI	Plaque Index
PPD	Probing Pocket Depth
PPE	Personal Protective Equipment
SARS-CoV-2	Severe Acute Respiratory Syndrome Coronavirus 2
SOFA	Sequential Organ Failure Assessment
STROBE	Strengthening the Reporting of Observational Studies in Epidemiology
TNF-α	Tumor Necrosis Factor-alpha
